# Energy Metabolism of the Brain, Including the Cooperation between Astrocytes and Neurons, Especially in the Context of Glycogen Metabolism

**DOI:** 10.3390/ijms161125939

**Published:** 2015-10-29

**Authors:** Anna Falkowska, Izabela Gutowska, Marta Goschorska, Przemysław Nowacki, Dariusz Chlubek, Irena Baranowska-Bosiacka

**Affiliations:** 1 Department of Biochemistry and Medical Chemistry, Pomeranian Medical University, Powstańców Wlkp. 72, 70-111 Szczecin, Poland; a.falkowska@pum.edu.pl (A.F.); rcmarta@wp.pl (M.G.); dchlubek@sci.pam.szczecin.pl (D.C.); 2 Department of Biochemistry and Human Nutrition, Pomeranian Medical University, Broniewskiego 24, 71-460 Szczecin, Poland; izagut@poczta.onet.pl; 3 Department of Neurology, Pomeranian Medical University, Unii Lubelskiej 1, 71-225 Szczecin, Poland; przemyslaw.nowacki@pum.edu.pl

**Keywords:** brain energy metabolism, astrocytes, brain, glucose, glycogen, neurons

## Abstract

Glycogen metabolism has important implications for the functioning of the brain, especially the cooperation between astrocytes and neurons. According to various research data, in a glycogen deficiency (for example during hypoglycemia) glycogen supplies are used to generate lactate, which is then transported to neighboring neurons. Likewise, during periods of intense activity of the nervous system, when the energy demand exceeds supply, astrocyte glycogen is immediately converted to lactate, some of which is transported to the neurons. Thus, glycogen from astrocytes functions as a kind of protection against hypoglycemia, ensuring preservation of neuronal function. The neuroprotective effect of lactate during hypoglycemia or cerebral ischemia has been reported in literature. This review goes on to emphasize that while neurons and astrocytes differ in metabolic profile, they interact to form a common metabolic cooperation.

## 1. Introduction

In most human tissues, glycogen is the stored form of glucose and performs various functions depending on the location in the body. At low blood glucose levels, the glycogen stored in the liver is metabolized into glucose that is subsequently released into systemic circulation; in this way hepatic glycogenolysis serves to control blood glucose. Glycogen also provides skeletal muscles with energy via glycolysis in response to increased energy demand, e.g., during intense exercise. The presence of glycogen in the brain, although lower than in the liver or muscles, indicates its essential role in neuronal activity [1].

Neuronal metabolic processes in the brain depend on the activity of astrocytes, which produce lactate and activate glycolysis and glycogen metabolism. Although the involvement of glycogen in maintaining neuronal activity in the brain is unquestionable, it is still widely debated to what extent energy derived from glycogen is consumed by the astrocytes themselves, and how much is directed to neurons in the form of lactate. It is also necessary to clarify the connection between glycogen metabolism and neuronal glutamatergic transmission.

## 2. Location of Glycogen in the Brain and the Metabolic Compartments of the Brain

The cellular localization of brain glycogen is highly specific. It is generally accepted that glycogen is found predominantly in astrocytes [2], although it has also been found in embryonic neurons [2,3].

In the adult brain, glycogen is found in astrocytes, although it is not clear whether levels are the same in all types of astrocytes [4,5], with subtypes being determined on the basis of morphology and functional characteristics.

The astrocyte end-feet may even cover the entire surface of the capillary. The end-feet show the presence of GLUT1 type glucose transporters and are sites of glucose uptake. Astrocytes, on one hand, can communicate with capillaries, and on the other are associated with neurons and synaptic processes. The metabolic brain concept is based on this integrated cooperation between astrocytes and neurons.

Brain glycogen is metabolized by enzymes located in astrocytes, such as glycogen phosphorylase (GP) and glycogen synthase (GYS) [6]. The brain isoform of GP occurs mainly in astrocytes, but interestingly it can also be found in several other cell types, such as the choroid plexus cells and ependymal cells [7]. GYS also occurs in neurons [8]. Complementary DNA (cDNA) for brain GYS is 96% homologous to the muscle isoform, and to a lesser extent to the hepatic isoenzyme [8]. Widely distributed throughout the brain, GYS is mainly expressed in the hippocampus, cerebellum and olfactory bulbs [8], occurring in both inactive phosphorylated (GYSb) and active dephosphorylated (GYSa) forms, which allows a precise regulation of glycogen metabolism. The transformations of phosphorylated/dephosphorylated synthases are controlled by a whole family of phosphatases [8].

Glycogen is not evenly distributed throughout the brain. Microscopic examinations show that the concentrations of glycogen are highest in regions with the highest synaptic density [9], suggesting its role in synaptic transmission, with the concentrations in gray matter about two times greater than in white matter [10]. High levels of glycogen can be found in the medulla oblongata, pons, cerebellum, hippocampus, hypothalamus, thalamus, cortex and striatum [9].

The energy metabolism of the brain is also associated with compartmentalization. In neurons and astrocytes there are compartments that can be characterized by specific conditions: e.g., synaptic vesicles (only in neurons). The cytoplasm is very heterogeneous, containing a high local concentration of metabolites, macromolecules and ions. Mitochondria are also metabolically heterogeneous. Through electron microscopy using a labeled α-ketoglutarate dehydrogenase (a key enzyme of the Krebs cycle), it has been shown that mitochondria in astrocytes in the same cell are distributed unevenly and have diverse potential, indicating differences in the ability of mitochondria to carry out oxidative metabolism [11]. This means that some of the mitochondria can be adapted to produce energy in the form of ATP, while others can perform other functions, e.g., anaplerotic (auxiliary) reactions related for example with glutamine synthesis and exporting it to the neuron as a precursor for glutamate and γ-aminobutyric acid (GABA). Mitochondria are very dynamic, constantly changing their number in astrocytes and the network that create.

## 3. Regulatory Mechanisms for Glycogen Metabolism and Uptake of K^+^ in Astrocytes

Glycogen is found in many different tissues, all with differences in glycogen metabolism. Glucose is transported to cells via several glucose transporters, reaching the brain through the glucose transporter GLUT1; within brain astrocytes and oligodendrocytes also via GLUT1 and microglial cells GLUT 5 [12], while neurons cells use GLUT3. Importantly, glycogen synthesis and degradation may take place simultaneously and are subject to complex regulation [13,14].

Although astrocytes are not excitable cells, they are responsible for a critical stage in the absorption of excess K^+^ released by neurons into the extracellular space during synaptic activity, at the time of generation of action potential [15,16,17]. Glycogenolysis and the uptake of K^+^ ions have been found to have a special mutual relationship in astrocytes [18]. It has been shown that in astrocyte cultures, decomposition of glycogen delivers energy driving the uptake of K^+^ ions [19,20]. Importantly, the uptake of ions by astrocytes ceased in the case of halting glycogenolysis and glycogen phosphorylase (GP) inhibition activity, and the actual activity of the brain appeared to be limited to just astrocytes.

The breakdown of glycogen by glycogen phosphorylase (GP) in the brain is controlled by both phosphorylation/dephosphorylation and allosteric mechanisms. Control performed by phosphorylation involves activation of phosphorylase kinase (PK), which then phosphorylates GP causing it to move from the normally inactive form (GPb) to the active phosphorylated form (GPa).

PK contains four Ca^2+^ binding sites. The increase in the level of Ca^2+^ in the period of increased uptake of K^+^ may result from the intracellular signaling cascade, but also may be caused by the increased activity of Na^+^/Ca^2+^ exchanger (NCX) proteins and/or plasma membrane L-type voltage-gated calcium channels (LCCs) [21]. The increase in the uptake of K^+^ also changes intracellular pH by increasing the flow of carbohydrate by the Na^+^/HCO_3_^−^ cotransporter (NBC) [22]. The increase in HCO_3_^−^ results in the stimulation of adenylate cyclase (AC) [19], which converts adenosine triphosphate (ATP) to cyclic adenosine monophosphate (cAMP). The subsequent binding of cAMP to cAMP-dependent protein kinase A (PKA) leads to immediate phosphorylation of PK. Thus, as has already been mentioned above, phosphorylase kinase alone (PK) is controlled by both covalent modifications and allosteric mechanisms.

The GPb form of the brain glycogen phosphorylase may also be allosterically activated by AMP, where the aforementioned binding triggers a conformational change in GPb from the tense conformation (T) into the relaxed conformation (R). The R form has similar catalytic properties to the GPa phosphorylated form. AMP can also stimulate 5′-AMP-activated protein kinase (AMPK), either allosterically or by inhibiting dephosphorylation. AMPK has a glycogen-binding domain (GBD) that promotes glycogenolysis after activation of AMPK (and halting glycogen synthesis at the same time) [23,24]. In turn, glycogen regulates the activity of AMPK as the allosteric inhibitor of kinase [25].

**Figure 1:**
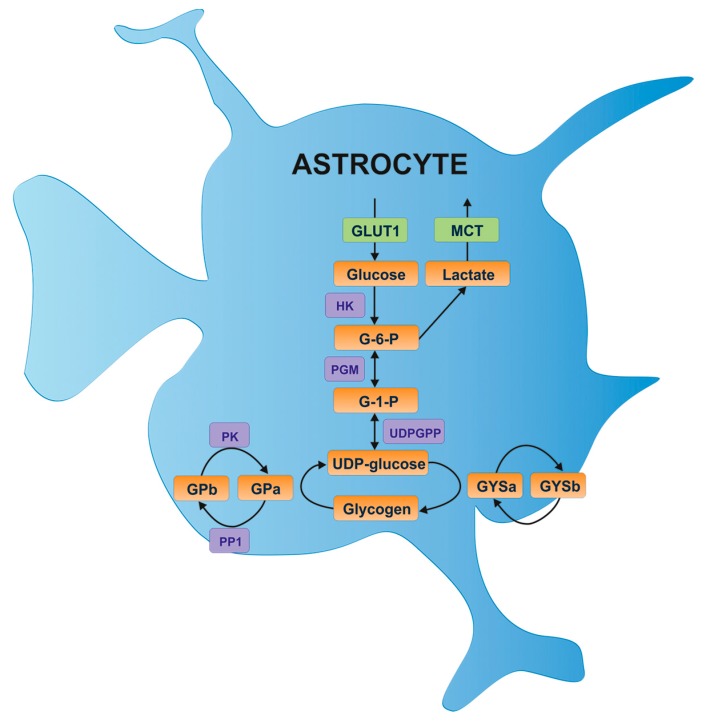
Glucose entry and glycogen formation in astrocytes [13]. Glucose is transported via the glucose transporter 1 (GLUT1) and possibly the insulin-sensitive glucose transporter 4 (GLUT4). Glucose is phosphorylated by hexokinase (HK) to glucose-6-phosphate (G-6-P), which is subsequently converted to glucose-1-phosphate (G-1-P) by phosphoglucomutase (PGM) and then to UDP glucose by UDP glucose pyrophosphate (UDPGPP). The UDP glucose continues on to glycogen synthesis via the actions of glycogen synthase (GYS), which can exist in two forms: the active dephosphorylated form (GYSa) or the inactive phosphorylated form (GYSb). Protein phosphatase 1 (PP1) converts GYSb to active GYSa via the regulatory subunit Protein Targeting to Glycogen (PTG), resulting in glycogen formation. Glycogen is broken down by glycogen phosphorylase (GP), which similar to glycogen synthase exists in two forms: the active phosphorylated form (GPa), or the inactive dephosphorylated form (GPb). Phosphorylase kinase (PK) dephosphorylates GPb to the active form. A major glycogen-derived product is lactate, which is transported into the extracellular space via monocarboxylate transporters (MCT).

Unlike GP, glycogen synthase (GYS) is active in the dephosphorylated form (GYSa) and inactive in the phosphorylated form (GYSb). However, the mutual regulation of GS and GP by covalent phosphorylation does not translate into mutually exclusive processes of synthesis and breakdown of brain glycogen. In the brain the simultaneous activation of GYS and GP takes place only under certain conditions. The fact that the balance of glycogen turnover at a steady state is not zero is probably due to the presence of different allosteric effectors. Although at the tissue level the rate of synthesis must be equal to the rate of degradation, individual glycogen molecules can be found at various points of metabolism (synthesis/degradation), so that the final rates of glycogen synthesis and degradation do not overlap [26,27]. Importantly, the glycogen synthase (GYS) and glycogen phosphorylase (GP) enzymes are the key subjects to the complex regulation of glycogen metabolism (Figure 1).

Recent studies also report that K^+^ ions affect the regulation of carbohydrate transport into astrocytes via Na^+^/HCO_3_^−^ cotransporter (NBC) [19,28,29,30,31,32,33]. The extracellular HCO_3_^−^ may be produced with the involvement of carbonic anhydrase IV (CA-IV), which is the predominant isoform in astrocytes [34]. Interestingly, an increase in intracellular HCO_3_^−^ results in the stimulation of adenylate cyclase and cAMP production, as well as the stimulation of glycogen breakdown. Thus, the involvement of bicarbonate-induced glycogenolysis adds to the pathways leading to activation of PKA and PK/GP phosphorylation cascade.

Glucose may be oxidized via the pentose-phosphate pathway (PPP), highly important not only for neurons but also for many other brain cells. This pathway contributes to the production of NADPH and 5-carbon sugar. It runs in two steps; first—the oxidative step (irreversible)—wherein NADPH is generated, and a second—the non-oxidative step (reversible)—where PPP is regulated by glucose-6-phosphate dehydrogenase (G6PD); the regulation occurs via NADPH:NADP^+^ ratio and the allosteric induction by NADP^+^. The normally high NADPH:NADP^+^ ratio makes the cell cytosol an environment rich in NADPH, a reducing agent required in the synthesis of lipids and nucleic acids. Therefore, PPP is of great importance in a developing brain compared to a mature brain, for example in terms of lipogenesis and myelination in the nervous system [35]. High PPP activity can also be found in the mature brain [35] where PPP-derived NADPH can be used for metabolizing neurotransmitters and gliotransmitters, as well as aldehydes and peroxides [35]. In this way, NADPH also promotes regeneration of glutathione (GSH).

Phosphofructokinase B3 (PFKB3) is the major isoform expressed in the brain. It has been shown that PFKB3 is continuously degraded by E3 ubiquitin ligase anaphase-promoting complex/cyclosome (APC/C)-Cdh1 [36]. Neurons are not able to enhance glycolysis with a lack of PFKB3. When mitochondrial respiration is inhibited, astrocytes reactivate their glycolytic properties through the production of fructose 2,6-bisphosphate (Fru-2,6-P_2_), whereas neurons have been shown to die rapidly in these conditions [35]. Therefore, glucose in neurons must be oxidized via other metabolic pathways, such as PPP.

**Figure 2:**
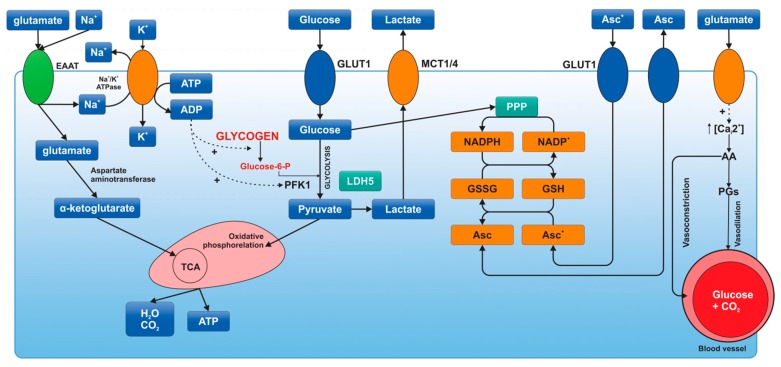
Metabolic activation of astrocytes [40]. Synaptic activity produces an increase in extracellular K^+^, which stimulates Na^+^K^+^-ATPase by binding of its extracellular K^+^-sensitive site. The excitatory neurotransmitter glutamate is taken up by astrocytes through excitatory amino acid transporters. This kind of transport produces an increase in intracellular Na^+^, which stimulates Na^+^K^+^-ATPase by binding of its intracellular Na^+^-sensitive site. Na^+^K^+^-ATPase activation produces a decrease in ATP/ADP ratio, thus glycolysis and glycogenolysis activation. In addition, glucose is oxidized by pentose phosphate pathway (PPP) to produce NADPH and to maintain the redox balance, reducing glutathione and ascorbic acid (Asc). Ascorbic acid released by astrocytes is taken up by neurons to protect themselves from oxidant species (ascorbic acid is oxidized in neurons). Oxidized ascorbic acid (dehydroascorbic acid, Asc^+^) is released from neurons and taken up by astrocytes through GLUT1. In astrocytes, glutamate is able to bind ionotropic receptors, which are predominantly calcium channels. This Ca^2+^ increase cooperates with Krebs cycle (TCA) activation and produces arachidonic acid (AA) and prostaglandin (PGs), which stimulate the constriction and dilation of capillaries, respectively. ADP: adenosine diphosphate; ATP: adenosine triphosphate; EAAT: excitatory amino-acid transporter; GLUT: glucose transporter; G-6-P: glucose 6-phosphate; GSSG: glutathione disulfide; GSH: glutathione; LDH: lactate dehydrogenase; MCT: monocarboxylate transporter; Na^+^K^+^-ATPase: sodium-potassium pump; NADP^+^: nicotinamide adenine dinucleotide phosphate (NADPH-reduced form of NADP); PFK1: phosphofructokinase-1; TCA: Krebs cycle.

Research has also shown that neuronal intracellular ascorbic acid inhibits the uptake of glucose in neurons by inhibiting GLUT3 [37,38]. On the other hand, intracellular ascorbic acid is able to stimulate the transport of lactate in neurons and cells that express GLUT3 [38]. Since ascorbic acid is able to change the preference for energy substrates, this mechanism is known as “the ascorbic acid metabolic switch” [37,39] (Figure 2).

As we can see brain glycogen levels are strictly regulated. Among other things, they depend on hormones such as adrenaline and noradrenaline, which have a glycogenolytic action, and insulin, promoting the synthesis of glycogen. Insulin-like growth factor-I and II (IGF I, IGF II) as well as insulin, may increase the levels of brain glycogen by their influence on insulin receptors [14].

## 4. Lactate as a Major Metabolite. The Hypothesis of Lactate Transfer between Astrocyte and Neuron

Until recently it has been believed that due to their high energy demands neurons synthesize energy primarily by the oxidative metabolism of glucose (Krebs cycle and respiratory chain) using glucose as fuel [17]. However, a lot of evidence suggests that neurons can efficiently utilize lactate and, in addition, have a preference for lactate if both glucose and lactate are present [17].

It has been shown that the enzyme phosphofructokinase B3, connected with the glycolysis input pathway for glucose, is practically absent in neurons because of its solid proteasomal degradation, whereas astrocytes show high levels of expression of this enzyme. It has also been demonstrated that neurons exhibit a slower rate of glycolysis as a result of low production of fructose-2,6-bisphosphate, which is the strongest activator of phosphofructokinase, a key enzyme of glycolysis, in contrast to astrocytes, which indicates an increased amount of activator and thus the rate of glycolysis.

Interestingly, excessive activation of glycolysis in neurons leads to oxidative stress and apoptosis of neurons. This suggests that neurons cannot afford to maintain a high rate of glycolysis. At the same time it has been shown that increasing the glucose concentration starts the hexose monophosphate pathway (HMP) in neurons. It follows that the balance between the glycolytic pathway and the HMP cycle must be strictly maintained in neurons to meet their energy needs, while retaining their antioxidant status. Accordingly, the use of lactate as a substrate may be a source of an oxidant to produce large amounts of ATP, apart from the glycolytic pathway, which can save neurons glucose for the HMP cycle, and thus protect neurons.

**Figure 3:**
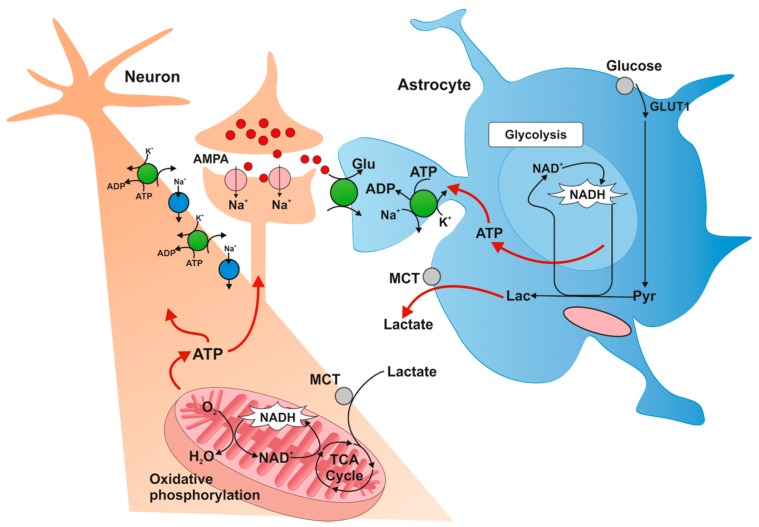
Metabolic profile of astrocytes [16]. Glucose is transported to astrocytes through GLUT1 transporter and then metabolized to lactate. Lactate is transported outside astrocytes and taken up by neurons by monocarboxylate transporters (MCTs). Intracellular lactate in neurons is oxidized to pyruvate and metabolized along the oxygen pathway. GLUT1: glucose transporter-1; MCT: monocarboxylate transporter; TCA: Krebs cycle; Pyr: pyruvate; Lac: lactate.

Astrocytes display a very high glycolytic activity (Figure 3). Although in comparison with neurons they have lower rates of oxygen metabolism, they very quickly metabolize glucose via the glycolytic pathway. The glycolytic nature of astrocytes and their preferences for the production and release of lactate are also conducive to the production of pyruvate, which is then included in the Krebs cycle.

It has been believed that the astrocytes cover 5%–15% of the energy needs of the brain. The evidence presented in recent years, however, suggests that the contribution of astrocytes in brain energy metabolism processes may have been underestimated.

It has been shown that in the rat brain at rest, astrocytes are responsible for the metabolism of around 50% of glucose absorbed by the brain, and this increases during activation of astrocytes. How, then, can be these data reconciled with the fact that neurons and not astrocytes have the greatest energy demand? How to explain this apparent paradox? The transfer of energy substrates from astrocytes to neurons provides a simple hypothetical explanation.

It has been shown that the activity of glutamatergic neurons increases the concentration of extracellular glutamate that is taken up by astrocytes using sodium-dependent glutamate transporters by increasing the concentration of Na^+^ in astrocytes. This in turn activates the Na^+^/K^+^ ATPase and thereby stimulates glucose uptake and glycolysis in astrocytes. Then it leads to increased production of lactate, which is released into the extracellular space, is obtained by neurons via monocarboxylate transporters (MCT) transporters [3] and then metabolized. This concept of *lactate transfer*, introduced by George Brooks [41], is not uncommon, nor limited only to astrocytes and neurons, and encompasses both intracellular, intercellular and intra-organ transfers of lactate.

As demonstrated, the uptake of glutamate by astrocytes increases the use of glucose and lactate release from the cells. Interestingly, after the exposure of cell cultures of neurons and astrocytes to glutamate stimulation, the stimulation of glucose utilization in astrocytes was concurrent with a rapid inhibition of glucose transport into the neurons, and was enhanced if lactate was present in the culture medium. Glutamate inhibited glucose transport into the neurons (and accordingly inhibited glycolysis), making it necessary for neurons to metabolize lactate as a substrate.

Glucose in these circumstances is used by neurons, as mentioned in the HMP pathway to produce NADP, which serves to protect neurons from reactive oxygen species.

**Figure 4:**
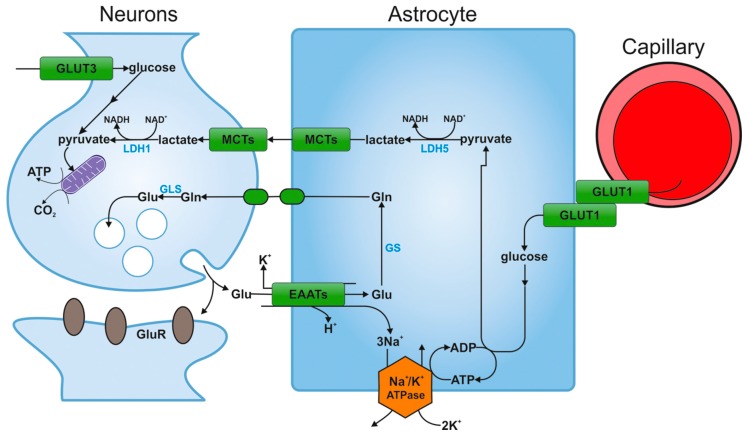
The hypothesis of astrocyte-neuron lactate shuttle [16]. Glucose is transported from blood vessels to astrocytes by endothelial cells. After entering the astrocyte via GLUT1, part of the glucose is metabolized to lactate via pyruvate by the isoenzyme of lactate dehydrogenase (LDH5). Then lactate is transported outside the astrocyte through the MCT transporter and is captured by neurons, also via the MCT transporter. Intracellular lactate in the neuron is oxidized to pyruvate by the other isoenzyme of lactate hydrogenase (LDH1) and is metabolized along the oxygen pathway. Glucose may be also transported directly into neuronal cells and penetrates into these cells via GLUT3. GLUT1, GLUT3: glucose transporters; MCT: monocarboxylate transporter; TCA: Krebs cycle; GS: glutamine synthetase; Glu: glutamate; Gln: glutamine; GluR: receptor for glutamate; EAATs: excitatory amino-acid transporters; GLS: glutaminase; LDH1, LDH5: isoenzymes of lactate dehydrogenase.

In recent studies in culture conditions, medium glucose deprivation caused a 50% inhibition of synaptic transmission in hippocampal neurons. However, the supply of glucose to astrocyte syncytium restored activity of the neurons. This effect was the same for the delivery of lactate, and did not occur in the presence of monocarboxylic acid transporter inhibitor which carries lactate to the neurons, which clearly confirms that astrocytes glucose is metabolized to lactate, which is then used by neurons to maintain their synaptic activity (Figure 4). Many studies support the idea of net energy transfer from astrocytes to neurons in the form of lactate as a result of glutamatergic transmission.

## 5. Glycolysis and Lactate Production Support Neuronal Activity

The lactate transfer shows that glycolysis and lactate are released place mostly in astrocytes, while lactate oxidation takes place in neurons [41]. Neuronal metabolism seems to be mainly oxidative and that astrocytic metabolism is glycolytic [42], even though astrocytes and neurons have the same oxidative capacity.

The transport of lactate across the blood-brain barrier is carried out by MCT1 [43,44]. At rest, the arterial blood concentration of lactate is <1 mmol/L, and increases during exercise [45]. Lactate is cotransported with H^+^ [46,47] and so the lactate transport is influenced by H^+^ (pH) gradient between blood and the brain; when pH becomes low, the lactate uptake increases [48]. Accordingly, when the brain is exposed to a considerable lactate load and a favorable H^+^ gradient, lactate uptake by the brain is significantly enhanced [49]. At an arterial lactate level higher than 3 mmol/L (during moderate exercise), the lactate uptake by the brain is quite low [50]. However, as a result of a maximal exercise during which arterial lactate rises to 15 mmol/L or higher, its uptake by the brain may be higher than that that of glucose [51,52,53,54].

Neuronal lactate uptake takes place mostly via MCT2, a high affinity isoform (low Vmax), while astrocytic uptake involves the expression of MCT1 and MCT4, low-affinity carriers (high Vmax) [55]. In the resting brain lactate concentration, uptake by neurons is 60% saturated and slower than the astrocytic uptake [56].

In exercises involving legs, higher cerebral activity appears to induce a higher cerebral lactate uptake than in exercises involving arms. In general, training increases MCT expression and thus enhances skeletal muscle capacity for lactate transport [57]. As exercise has also been found to upregulate a few neuronal genes [58], including motorcortex oxidative capacity [59], it may be speculated that neurons and astrocytes are also able increase their capacity for lactate transport. Moreover, at diverse intensities of exercise, lactate metabolism in the cortex appears be higher in well-trained subjects than in control [60] and could be significant for shaping exercise capacity.

During a 1 h long recovery period, the lactate taken up by the brain during exercise is neither released to the blood [61,62], nor accumulates in the cerebrospinal fluid (CSF), nor in the brain [63]. As the extracellular lactate pool receives lactate from glycolysis and glycogenolysis, the lactate is metabolized, transported to brain regions separate from the region of interest, or might be used for synthesis. When it comes to lactate used as a biosynthesis precursor, astrocytic and neuronal gluconeogenesis is very low [64]; glycogen synthesis from lactate does not appear to be significant, either [65,66]. The glucose sparing effect of lactate could be explained by the fact that glycolysis may be inhibited by the conversion of lactate to pyruvate and the energy metabolites of the subsequent oxidation [67,68,69,70,71]. Therefore, it is still not clear if lactate is indeed preferred over glucose. It is possible that the oxidation of lactate to pyruvate is favorable in low energy status, as it does not need ATP for activation.

In conclusion, intense exercise increases the level of arterial lactate decreases the pH and the brain becomes increasingly activated, which all contribute to higher cerebral lactate uptake. In these circumstances, lactate metabolism in the brain may reach 50% of glucose metabolism; together, both fuel brain functioning under exercise [72]. The lactate taken up by the brain is neither released from the brain nor accumulated there, and so is probably metabolized [73].

Neuronal-astrocytic coupling mechanisms are also crucial for memory formation [74]. In a study on rats, Suzuki *et al.* found that learning leads to a significant increase in hippocampal extracellular lactate levels derived from astrocytic glycogen [74]. The long-term memory (although not short-term memory) depends crucially on astrocytic glycogen breakdown and lactate release. Interestingly, when the expression of the astrocytic lactate transporter MCT4 or MCT1 is disrupted, the resultant amnesia is rescued by lactate but not glucose. In the case of the disrupted expression of the neuronal lactate transporter MCT2, neither lactate nor glucose may counteract amnesia, which again indicates that long-term memory critically depends on lactate uptake by neurons [74].

## 6. The Key Role of Astrocytes in Neurotransmission and the Importance of Brain Glycogen in Glutamatergic Transmission

Some relationships occurring between astrocytes and other parts of the nervous system are particularly important in the discussion of brain energy metabolism at the cellular level. Processes associated with astrocytes are focused around synapses, especially in the so-called end-feet surrounding the capillaries of the neural tissue and serving to ensure the proper relationship between the blood and other parts of the brain.

**Figure 5:**
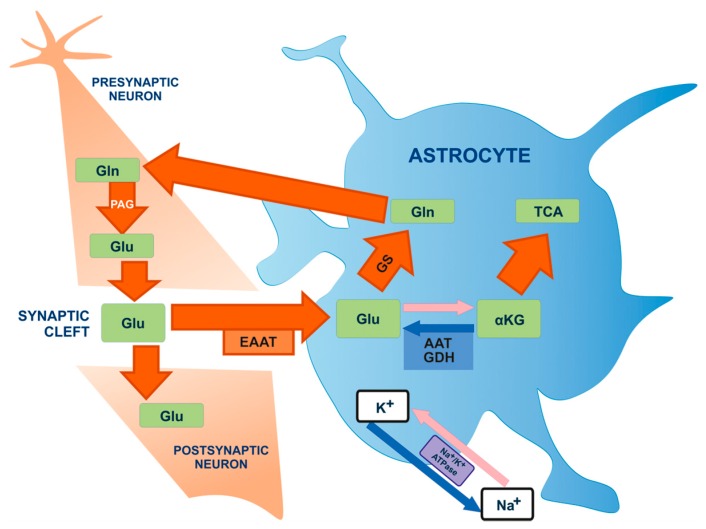
Glutamate-glutamine cycle [77]. Glutamate (Glu) molecules are released from the pre-synaptic neuron into the synaptic cleft where they can stimulate glutamate receptors on the post-synaptic neuron. Glutamate also penetrates the synaptic gap towards astrocytes. Glu is mainly transported to astrocytes by Na^+^-dependent excitatory amino-acid activating transporters (EAATs). This leads to the occurrence of astrocyte ion gradients of Na^+^, which stimulates Na^+^/K^+^ ATPase in order to restore proper ionic concentrations. Glutamate is transformed into: glutamine (Gln) by means of glutamine synthase enzyme (GS) or to α-ketoglutarate (α-KG) by means of glutamate dehydrogenase (GDH) or aspartate aminotransferase (AAT), in order to conduct further oxygen metabolism in the TCA cycle (Krebs cycle). Glutamine is transferred to neurons in order to conduct further glutamate production with the participation of phosphate-activated glutaminase (PAG).

The rapid removal of neurotransmitters released into the synaptic cleft is one of the best-known roles of astrocytes. This is particularly important in the case of glutamate, the most important excitatory neurotransmitter in the brain, due to the highly excitotoxic effect of over-stimulated glutamate receptors on neurons. Glutamate uptake occurs mainly through astrocyte-specific glutamate transporters. In addition, glutamate is also transferred back to the neurons via the glutamate-glutamine cycle (Figure 5), in which glutamine synthetase converts astrocytic glutamate to glutamine, which is then transported to neurons. There, it is transformed back to glutamate in a deamination reaction [75,76]. Thanks to this cycle, astrocytes are involved in replenishing the pool of glutamate in the brain, and the effect of glutamate as a neurotransmitter in the synapse is strongly dependent on astrocyte metabolism [14].

CNS cells exhibit a strong functional link, with the ability to transmit signals conditioned by the close proximity of astrocytes and neurons. The information flow occurring during the cooperation between astrocytes and neurons is associated with the release of various messenger molecules. These include factors stimulating the growth and differentiation of nerve cells, cytokines, and neurotransmitters regulating the functionally mature nervous system. Neurons are 100 times more sensitive to glutamate than astrocytes, and hence the significance of astrocytes in regulating the Glu concentration. Glutamate transport into the astrocyte occurs according to the concentration gradient. Astrocytes bind Glu released by the neurons into the synaptic cleft via conveyors on their surface. Then, with the use of Na^+^/K^+^-ATPase and the energy stored in ATP they transport it into the cell. The absorbed Glu is then converted to glutamine by glutamine synthetase (an enzyme not present in nerve cells). The resulting Gln is directed to neurons via Na^+^-dependent transporters of amino acids and is included in the series of conversions leading to the formation of active Glu, which occur with the participation of neuronal mitochondrial glutaminase [75,76].

A study conducted by Mozrzymas *et al.* 2011 compared the results of inhibiting the effect of glycogen breakdown on miniature excitatory postsynaptic currents (mEPSCs) in pure neuronal cultures (PNC) of rat hippocampal neurons and astrocyte-neuronal co-cultures (ANCC) [78]. The amplitudes of mEPSC in ANCC were almost twice as high as in PNC, without significant differences in the reaction kinetics [78]. The inhibition of glycogen phosphorylase reduced mEPSC amplitude by about 40% in ANCC, but had no effect on PNC. This indicates that the astrocyte-neuron interactions reinforce the primary mEPSCs in ANCC, thanks to astrocytic glycogen metabolism [78]. The amplitudes of mEPSCs in the ANCC were almost two times higher than in the PNC, and most of those differences were cancelled by glycogen phosphorylase inhibitor (BAY U6751), ineffective in the case of PNC [78]. Although the impact of glycogen phosphorylase on the regulation of mEPSCs processes is visible, the precise mechanism is not yet understood. Most probably, the activity of glutamatergic synapses is associated with a variety of compounds linked with astrocytes whose effect on the neuronal activity is dependent on glycogen phosphorylase. Factors potentially involved in these mechanisms include lactate, Gln, Glu (both neuronal and astrocytic), ATP, neurotrophins, *etc.* [79]. However, further studies are needed to clarify the participation of these factors in regard to the relationship between astrocytes and neurons.

Synaptic plasticity (especially glutamatergic synapses) is believed to be a major substrate for memory and learning. More and more evidence suggests the crucial role of astrocyte glycogen metabolism in the long-term regulation of neuronal activity. A recent study by Hertz and Gibbs showed the critical role of astrocytic metabolism in memory consolidation, which depends on glutamatergic synaptic plasticity [80]. The basal synaptic transmission is an important feature, characterized by the readiness of the neural network for an adequate response. It has been shown that glycogen is able to maintain neuronal activity even in energy deficient conditions or in other pathological conditions [81]. In summary, normal glycogen metabolism is essential for glutamate neurotransmission, and the energy generated during the degradation of glycogen is necessary for the performance of astrocytes and neurons [81].

## 7. The Effect of Hypoglycemia on Brain Energy Metabolism

Many reports suggest that human brain glucose metabolism changes under hypoglycemic conditions, yet the exact changes are unclear [82].

Hypoglycemia may influence the cognitive and memory processes that are associated with changes in the energy metabolism of the brain. Despite the high sensitivity of neurons to hypoglycemic states, certain neurochemical changes allow the cells to become resistant to low concentrations of glucose [83].

Due to a slow glycogen metabolism at rest and a rapid mobilization during energy crisis or hypoglycemia [83,84], glycogen is considered to be the main emergency energy substrate [85,86]. Its physiological role also includes effective support of brain activity when glucose cannot meet the high energy demands [85,87,88,89]. Lactate derived from glycogen metabolism is able to maintain neuronal function in the absence of glucose [90]. Consciousness disorders associated with hypoglycemia are characterized by an inability to detect hypoglycemia, which may result from the limited availability of alternative energy substrates, and reduced activity of glucose itself [83,91]. If the concentration of intracellular glucose stores (*i.e.*, glycogen) increases during hypoglycemia, this might contribute to the masking of an increased demand by other cells for glucose, thereby causing hypoglycemia-related disturbances in consciousness [92]. This condition is associated with hypoglycemic autonomic failure and is very dangerous as there are no symptoms of hypoglycemia before the onset of cognitive impairment, which in turn can lead to sudden hypoglycemia and a coma [90]. Glycogen supercompensation, *i.e.*, an increase in glycogen content after hypoglycemia, provides evidence of the mobilization of brain glycogen during hypoglycemia [15].

Normal cerebral glucose metabolism during hypoglycemia may be maintained by several potential mechanisms. One possible explanation is a compensatory increase in the cerebral uptake of lactate. As plasma lactate increases by about 50% in response to hypoglycemia [93], it may be used by the brain as an alternative source of energy and reduce need for glucose in the brain [94,95,96,97]. When converted to pyruvate, lactate carbons may enter the TCA cycle, similar to glucose carbons [98]. Mason *et al.* (2006) indicated that during hypoglycemia the levels of transporters for monocarboxylic substrates such as lactate can increase two times, which is consistent with the proposition that lactate consumption increases during hypoglycemia [99,100]. Moreover, even under resting conditions, increased lactate availability stimulates its consumption by the brain while glucose utilization decreases.

A few recent *in vivo* studies have shown the neuroprotective effect of lactate during hypoglycemia or cerebral ischemia episodes; the brain preferentially utilized lactate over glucose, and so was able to sustain the activity of neurons for hours, even without glucose [101]. According to some papers, neurons are the preferential site of lactate oxidation, while lactate itself is produced by astrocytes. Neuronal activity *in vivo* in the presence of lactate as the primary energy source was first presented by Wyss *et al.* (2011) [102], whose results show that the decrease in glucose utilization depends on the level of brain activation and not only on blood lactate concentrations.

Although the lactate in the brain was long considered a sign of cerebral harm and hypoxia [103], lactate is now postulated to have physiological functions in the CNS. It seems to be an important element in the metabolic cooperation between neurons and glia cells, especially during increased brain activity [104,105]. In addition, in order to coordinate brain activity and energy supply, lactate levels are detected by a specific type of neurons (orexin neurons in the lateral hypothalamus) [106,107,108,109].

Glucose is used by neurons to maintain their antioxidant status via the pentose phosphate pathway (PPP) that cannot be fueled by lactate [110,111]. In the case of low plasma glucose levels and low concentration gradient, glucose transport into neurons is not sufficient [112] to stimulate their antioxidant PPP, and some neurons, especially vulnerable to reactive oxygen and nitrogen species, may not completely avoid oxidative damage. Glucose is also needed by the astrocyte to pump glutamate and as such plays a significant role in functional neuroenergetics [113].

Lactate is transported by MCTs in a cotransport with protons [114]. An increased lactate concentration may bring about changes in lactate influx and then in intracellular and extracellular pH in neurons. These changes in the proton gradient could interfere with nerve conduction and lead to a delayed response.

The extracellular lactate concentration seems to increase during stimulation [115] and there is also some evidence for a translocation of MCT2 to the membrane surface during stimulation [116]. This process would increase the transport of lactate and result in a higher intracellular lactate concentration. The increase in the conversion of lactate to pyruvate is enhanced by an increased lactate/pyruvate ratio; the lower pyruvate level results from a decreased glycolytic flux.

As shown by experiments on cell cultures, glutamate inhibits neuronal glucose uptake [117,118] and that upregulation of glycolysis under stress conditions is prevented [119]. Recently, it has also been found *in vivo* that increased neuronal firing mediates inhibition of glucose transport in neurons while stimulating astrocytic glucose uptake [120].

Given that the stores of intracerebral glycogen are limited and consumed in the absence of exogenous glucose within a few minutes [121], gluconeogenic activity in the brain is negligible [122]. It is therefore evident that neurons must rely on lactate as an energy substrate, especially under hypoglycemic conditions. Even in normoglycemia, Wyss *et al.* observed an increased turnover of lactate during increased activation [102].

In conclusion, lactate is capable of maintaining neuronal integrity in glucose deficiency. Furthermore, lactate is preferred over glucose if both these substances are available. Lactate is easily metabolized and its metabolism is activity-dependent. The results of van de Ven *et al.* (2011) indicate that in cerebral glucose metabolism is not affected by moderate hypoglycemia and there is no evidence for an association between moderate hypoglycemia and a reduced cognitive function [123]. Therefore, the healthy human brain seems to be able to withstand moderate reductions in plasma glucose, probably thanks to an increased availability of lactate.

## 8. The Effect of Hypoglycemia on Cerebral Blood Flow (CBF)

CBF rises when the glucose supply to the brain is limited by hypoglycemia or glucose metabolism is inhibited by pharmacological doses of for example 2-deoxyglucose (DG) [124].

A number of studies have examined the effects of insulin-induced hypoglycemia on CBF, some of which reported small or no changes [124,125,126], but most found significant increases in CBF [127,128,129]. The discrepant results may have been due to species differences, the presence or absence of anesthesia, the nutritional state of the animals, the level and duration of hypoglycemia, or differences in the methods employed to determine CBF [127,128,129].

The exact mechanism responsible for the increased blood flow is still being discussed. A vasodilator agent derived from vascular endothelium, the endothelium-derived relaxing factor (EDRF), has been shown to mediate the vasodilator effects of acetylcholine [130]. This factor has been identified as nitric oxide (NO) [131,132]. NO is produced in vascular endothelium and in neurons and glia by oxidation of arginine catalyzed by the enzyme nitric oxide synthase. Inhibition of NO synthesis reduces cerebral blood flow throughout the brain despite an elevation in mean arterial blood pressure (MABP) [133,134]. Inhibition of NO synthase activity has also recently been reported to attenuate the increases in CBF during insulin-induced hypoglycemia in anesthetized, mechanically ventilated piglets, suggesting that NO plays a role in mediating the cerebrovascular response to hypoglycemia [135].

In anesthetized, paralyzed and artificially ventilated rats, local CBF was markedly increased in most regions of the brain during insulin-induced hypoglycemia [136]. In a study in the late 1980s, Bryan *et al.* (1987) found that moderate hypoglycemia in awake restrained rats increased local CBF in some regions of the brain (e.g., in the cerebral cortex and basal ganglia but not in the hypothalamus and cerebellum); more severe hypoglycemia (plasma glucose level, 1.5 mM) caused even greater increases in local CBF throughout the brain, including the hypothalamus and cerebellum [136]. The results of recent studies demonstrate that hypoglycemia increases CBF in almost all regions of the brain, but the effect is strongly dependent on the degree of hypoglycemia. The level of hypoglycemia at which a marked increase in CBF occurs is similar to the level at which glucose consumption in the brain becomes moderately depressed [137,138] and ATP levels in the brain begin to fall [139].

Glucose transport across the blood-brain barrier becomes the rate-limiting step in the regulation of brain function when brain glucose concentrations approach zero, for example, during hypoglycemia [140,141] and it causes CBF to increase significantly [142]. Cerebral blood flow rises during hypoglycemia have been reported in several studies [143,144]. The increase in CBF during hypoglycemia can be considered neuroprotective because it represents an attempt to increase capillary glucose concentration for improved glucose supply to the brain when circulating glucose levels are below a critical level.

## 9. The Role of Other Alternative Energy Substrates for the Brain

In the absence of glucose, the brain has alternative energy sources, including ketones derived from fatty acid metabolism taking place mainly in the liver. These include 3-β-hydroxybutyrate (3BHM), acetoacetate, and acetone [145]. Ketones play a significant role especially during the maturation of the brain and deliver 30%–70% of the required energy to the immature brain [15]. The concentration of ketones in the brain is regulated by their concentration in the blood and the permeability of the blood-brain barrier, which depends on the number of MCTs [146]. It has been demonstrated that the immature brain of rodents has a high expression and activity of MCTs in comparison to the mature brain, which makes it possible to uptake 3BHB and acetoacetate, and use them efficiently for the production of energy, or synthesis of amino acids or lipids [15]. β-Hydroxybutyrate is metabolized primarily in neurons and is converted to glutamate and glutamine [145]. Ketone bodies are metabolized to acetyl-CoA, then transferred to the Krebs cycle (TCA) and oxidized in an amount sufficient to satisfy the high metabolic requirements of the brain. In the mature brain, blood ketone body levels are usually low and grow mainly as a result of prolonged fasting or a high-fat diet [146]. It is believed that ketone bodies are able to provide two thirds of the total energy required for the brain during starvation [145]. Furthermore, they significantly save the resources of glucose, as they can inhibit its oxidation, probably via the inhibition of pyruvate dehydrogenase complex (PCD) [147]. In this way, a certain amount of glucose can be maintained during prolonged fasting, and metabolized via glycolysis.

The presence of other non-glucose substrates such as pyruvate or α-ketoglutarate, which can be metabolized without the presence of a cytosolic NAD, can preserve the viability of cells. It has been shown that they significantly reduce pyruvate hypoglycemic neuronal death and improve the cognitive function of the brain [148]. This indicates the ability of neurons to adapt to a condition of reduced blood glucose. These metabolic changes allow neurons to maintain synaptic activity and maintain an appropriate level of ATP for a long time. Some of these non-glucose substances have a neuroprotective effect during hypoglycemia, revealing promising therapeutic strategies for patients with frequent episodes of hypoglycemia.

## 10. Diabetes and Energy Metabolism of the Brain—The Impact of Diabetes on Glycolysis, the TCA Cycle and the Metabolism of Glutamate

Glucose metabolism has important implications for the functioning of the brain. In the event of disturbances, e.g., in diabetes mellitus, conditions of glucose delivery to the brain may become disrupted.

Diabetes is a complex metabolic disorder characterized by a relative or absolute deficiency in insulin secretion and/or its influence. Chronic hyperglycemia in diabetes is associated with abnormalities in many organs, for example microvascular and macrovascular changes in the brain. In diabetes both hypo- and hyperglycemia may contribute to microangiopathy and its complications. Diabetes mellitus (types 1 and 2) is associated with an increased risk of cognitive impairment and dementia [149,150]. Patients with diabetes are at increased risk of developing Alzheimer’s disease and are 2–3 times more likely to have a stroke independently of other risk factors [151].

The significance of brain glycogen is becoming increasingly evident both in normal and pathological states. Unfortunately, it is not entirely understood how diabetes-induced brain disorders are associated with brain energy and neurotransmission. In this context, research should focus on the role of brain glycogen in the support of glycolysis and the TCA cycle, as well the glutamate-glutamine cycle in type 1 and 2 diabetes.

Diabetes is a complex metabolic syndrome that disrupts both signaling and metabolic pathways, which in turn affect the transport of glucose [144]. It is generally believed that the transport of glucose into nerve cells and astrocytes is independent of insulin; however, immunohistochemical studies have shown that insulin-sensitive glucose transporters GLUT4 and GLUT8 are present in some areas of the brain, which indicates that in a state of diabetes they may have an effect on the uptake of glucose [152]. The predominant transporter proteins (GLUT) involved in cerebral glucose utilization are GLUT1 and GLUT3, with GLUT1 present in all brain cells including the endothelial cells of the capillaries (with very low neuronal expression *in vivo*), and GLUT3 almost restricted to neurons [153]. GLUT1 is mainly responsible for the facilitative transport of glucose across the BBB. The BBB, which was considered to behave as a single transport step, separates the blood circulation compartment from the brain aqueous phase that is virtually separated from the metabolic pool where glucose is consumed [154]. The transport of glucose to the brain is downregulated in chronic hyperglycemia as well as during experimentally-induced hyperglycemia [144]. Changes in the transport of glucose to the brain during extreme hyperglycemia occur with no changes in the expression of insulin-sensitive mRNA or insulin insensitive transporters, while at the protein level insulin-insensitive glucose transporter GLUT1 is downregulated [155,156,157].

The activity of glycolysis differs between type 1 and type 2 diabetes [158]. Some data show that glycolytic activity is reduced in type 1 diabetes [157], while other studies show that at least initially glycolytic activity increases to a certain extent. The fact that glucose utilization initially increases suggests an increase in the oxidative metabolism of glucose through the TCA cycle [157]. In contrast, in type 2 diabetes, TCA cycle activity is reduced. One of the subunits of the PDC enzyme complex is downregulated [159].

Lactate concentration is reduced in the cerebral cortex in type 2 diabetes, in connection with the inhibition of glycogen degradation [144]. Lower amounts of lactate are also due to the presence of a glycogen phosphorylase inhibitor [144]. Glycolysis is activated in the presence of a glycogen phosphorylase inhibitor [160].

As mentioned, glutamate is an important intermediate in energy metabolism, e.g., linking the TCA cycle and amino acid metabolism. Due to the fact that Glu also acts as an excitatory neurotransmitter, its metabolism and regulation are particularly important. Glutamate homeostasis is related to the astrocyte-neuron relationship. Glu is released from presynaptic nerve terminals and interacts with receptors in the postsynaptic membrane, with astrocytes regulating the content of Glu in the synaptic cleft by influencing the glutamate transporters with high affinity located in the membrane of the astrocytes near the synaptic cleft. In order to maintain homeostasis, astrocytes return the glutamate precursor—glutamine—to the neurons, ending the glutamate-glutamine cycle.

Neurons are not able to generate intermediates of the TCA cycle or glutamate from glucose, which shows the importance of a continuous supply of precursor compounds from the astrocytes. Glycogen from astrocytes may contribute to *de novo* synthesis of glutamate. Processes related to the glutamate-glutamine cycle affect diabetes. As studies with rats show, glutamate uptake is increased in type 1 diabetes, and insulin treatment can normalize its uptake [161]. In the rat models, the activity of glutamine synthetase, which converts Glu into Gln in astrocytes, is increased in type 1 diabetes [162] and on the other hand, in type 2 diabetes glycogen phosphorylase inhibitors contribute to a significantly reduced amount of glutamate [163].

Summing up the impact of diabetes on metabolic processes, the brain appears to use compensatory mechanisms to ensure an adequate supply of glucose in a diabetic state. However, studies indicate that glucose transport itself and cellular metabolic interactions are weakened [144]. In addition, diabetes is associated with an alteration of both turnover and the activity of key enzymes involved in glycogen metabolism. For example, the amount of newly synthesized glycogen is reduced in type 1 diabetes, suggesting that the activity of glycogen synthase may be decreased [164]. Besides, energy homeostasis and homeostasis associated with neurotransmission may be disrupted.

## 11. Diabetes and Hyperglycemia

Chronic hyperglycemia may results in cerebral metabolic alterations and CNS injury. Uncontrolled chronic hyperglycemia, resulting from absolute insulin deficiency (type 1 diabetes) or insulin resistance without or with insulin deficiency (type 2 diabetes), is one of the leading causes of diabetic complications in a number of organs [165]. Hyperglycemia-induced (or associated) metabolic and vascular disturbances are known and may increase the risk of strokes, seizures, diabetic encephalopathy and cognitive compromise [166]. These pathological conditions may result from alterations in cerebral energy homeostasis and metabolism possibly through mechanisms including changes in osmolar gradients in hyperglycemia [167], hormonal regulation [168], glucose utilization [169], oxidative stress [170] and the levels of ketone bodies [171].

The aim of the study by Wang *et al.* (2012) showed that the onset of hyperglycemia in STZ-induced diabetes in an animal model of type 1 diabetes significantly increased brain glucose levels and altered the concentration of a number of neurochemicals related to osmotic regulation (e.g., GPC (glycerophosphorylcholine), Ins-myo-inositol, Tau (taurine), ketone bodies e.g., β HB (β hydroxybutyrate) [172,173]). As hyperglycemia persisted over 4 weeks, levels of additional neurochemicals including GSH and NAA were altered, which suggests increased oxidative stress and deterioration of neuronal integrity.

Hyperglycemia lead to osmolar gradients across cell membranes, triggering alterations in cell volume regulation that shift water from the intracellular fluid space to the extracellular fluid. The osmolar gradients due to the elevated glucose levels in hyperglycemia may have led to increased levels of intracellular osmolytes including GPC, Ins and Tau to promote the maintenance of neuronal and glial cell volume. Glycemic normalization was able to restore the GSH level but not that of NAA, indicating potential irreversible neuronal damage due to prolonged hyperglycemia.

## 12. Conclusions

To summarize the current evidence, this review shows that brain energy metabolism involves more complex mechanisms than were previously thought. Interactions between astrocytes and neurons have the purpose of not only meeting the energy needs of these cells, but are also important in the control of many important brain functions such as homeostasis of the body or memory consolidation. Metabolic disorders in the interactions between neurons and astrocytes can cause neurodegenerative disorders and a number of other pathologies. New therapeutic options in order to preserve or strengthen the neuroprotective function of astrocytes may set new directions for research.
